# Schizencephaly and Psychosis: A Rare Association

**DOI:** 10.1155/2013/210868

**Published:** 2013-12-17

**Authors:** Matias Carvalho Aguiar Melo, Saulo Giovanni Castor Albuquerque, José Henrique Sousa Luz, Alexandre Bastos Lima

**Affiliations:** ^1^Hospital de Saúde Mental Professor Frota Pinto Rua Vicente Nobre Macedo, S/N, Messejana, 60841-110 Fortaleza, CE, Brazil; ^2^Universidade de Fortaleza, Av. Washington Soares, 1321, Edson Queiroz, 60841-110 Fortaleza, CE, Brazil

## Abstract

Schizencephaly is a rare malformation of the central nervous system defined as a gray matter-lined cleft filled with cerebrospinal fluid that extends from the pial surface to the ventricle. Few cases of association with psychosis were reported in the scientific literature. We present a case of a 46-year-old woman, admitted into a psychiatric hospital with crises of psychomotor agitation, disorganized and erotized behavior, persecutory and self-reference delusions, and auditory and visual hallucinations. She also reported seizures since her childhood. A head CT scan revealed a large subarachnoid space communication with the adjacent lateral ventricle in the topography of occipital, temporal, and parietal lobes to the right, suggestive of schizencephaly.

## 1. Introduction

During recent years, a considerable amount of studies has addressed the association between diseases of the Central Nervous System (CNS) and the development of mental disorders. However, they have not received much importance and have required further investigations.

Malformations of central nervous system are often associated with neurological and psychiatric symptoms, such as mood and psychotic syndromes. Schizencephaly is a rare congenital disorder defined as a gray matter-lined cleft filled with cerebrospinal fluid that extends from the pial surface to the ventricle [1]. Correlations between that malformation, seizures, and developmental delay are well described.

However, few cases of psychosis associated with schizencephaly were reported in the scientific literature. Pubmed search with “Schizencephaly AND Psychosis” as Mesh terms resulted in 24 results. Of these only four discussed Schizencephaly. The rest of the twenty reports were related to other malformations.

## 2. Case Presentation

We present a case of 46-year-old woman, admitted into a psychiatric hospital with crises of psychomotor agitation, disorganized and erotized behavior, persecutory and self-reference delusions, and auditory and visual hallucinations. The episodes seem to have begun when she was an adolescent. The patient believed that she was medium and had a gift to see and to talk with spirits of dead people. She was not afraid of the souls, prayed enough for them and lighted candles. Nevertheless, her family, neighbors, and friends did not share her conviction.

Furthermore, the patient became suspicious and aggressive, thought other people had spoken badly of her, and broke objects for no apparent reason. During the crises, she closed the eyes, screamed, shook the head side to side, ran the hands through the body, and touched the genitals repeatedly. In these episodes, she claimed to be in another place and time and confused the hospital staff around her as distant relatives. While not having crises, the patient exhibits intelligence below expected levels, allopsychic disorientation, and left hemiparesis.

Additionally, there are reports of generalized tonic-clonic seizures since her childhood, often associated with trauma, tongue biting, and sphincter incontinence, under control with medication. An electroencephalography was performed which did not show any alterations. A video electroencephalography was not done due to the unavailability in local public service.

During physical examination, the patient was thin, disoriented in time, and cooperative. The walking was shaky and slow. Paresis of the left leg was detected. No abnormalities were found in heart and lung auscultation and abdominal examination.

**Figure 1:**
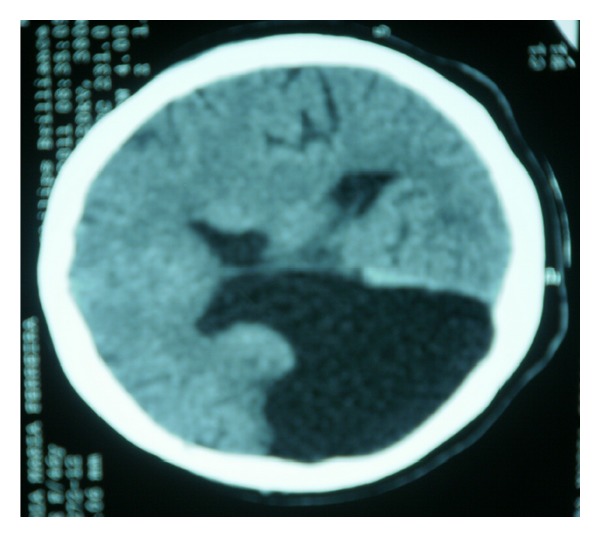
Head CT scan (October 4, 2011).

Examinations of blood count, biochemistry, and serology for HIV, syphilis, and hepatitis B and C showed results within normal limits. A head CT scan was also ordered, which revealed a large subarachnoid space communication with the adjacent lateral ventricle in the topography of occipital, temporal, and parietal lobes to the right, suggestive of schizencephaly (Figure 1).

Before the admission, she was treated with phenobarbital 100 mg/d, haloperidol 5 mg/day, biperiden 2 mg/day, and fluoxetine 20 mg/day. These last two medications were discontinued, the haloperidol dose was increased to 15 mg/day, and the barbituric was replaced by valproic acid 1 g/day. After almost 50 days in hospital, she got better in part and was discharged. An appointment was scheduled, but the patient did not attend.

She has a history of poliomyelitis at age 2. She is unaware of her pregnancy, childbirth, neuropsychomotor development, and level of education; however she does remember learning how to read, write, and make calculations with much difficulty. There are reports of several prior psychiatric admissions and an important family history for mental disorders.

## 3. Discussion

Schizencephaly could be the result of abnormal neuronal migration which occurs in the first half of the first trimester, although a few scholars state that such alterations are formed only in the early second trimester [2]. Nevertheless, infectious injuries and toxins may be associated with this condition [3].

The diagnosis of unilateral right schizencephaly (open lip) may justify the symptoms of psychosis, epilepsy, mental retardation, and possibly hemiparesis that we observed in this patient. The early onset of symptoms and partial response to treatment lead to this hypothesis. However, hemiparesis could be a consequence of a prior poliomyelitis infection during childhood.

Given the history of seizures which precede psychosis, an alternative diagnosis is a psychotic disorder caused by epilepsy. The family history of mental disorder may have an influence over her psychosis. The rupture of intracortical connections caused by schizencephaly may have worked as a mechanism for psychosis development in this patient. The theory that some psychoses are associated with abnormal brain development is becoming widely accepted. A possible association between psychosis and schizencephaly in this patient may provide an example of a neurological development abnormality that manifests itself as psychosis after a long delay [2].
